# Local problem solving in the Portuguese health examination survey: a mixed method study

**DOI:** 10.1186/s13690-022-00939-7

**Published:** 2022-08-24

**Authors:** Heidi Lyshol, Ana Paula Gil, Hanna Tolonen, Sónia Namorado, Irina Kislaya, Marta Barreto, Liliana Antunes, Vânia Gaio, Ana João Santos, Ana Paula Rodrigues, Carlos Matias Dias

**Affiliations:** 1 Department of Health and Inequality, Norwegian Institute of Public Health, Oslo, Norway; 2 CICS.NOVA - Interdisciplinary Center of Social Sciences, NOVA University of Lisbon, Lisbon, Portugal; 3 Finnish Institute for Health and Welfare, Helsinki, Finland; 4 Department of Epidemiology, National Institute of Health Doutor Ricardo Jorge, Lisbon, Portugal; 5 Public Health Research Center, National School of Public Health, NOVA University of Lisbon, Lisbon, Portugal; 6 Comprehensive Health Research Center, NOVA University of Lisbon, Lisbon, Portugal

**Keywords:** Health examination survey, Participation rate, Organizational improvisation, Procedure manual

## Abstract

**Background:**

Participation rates in health surveys, recognized as an important quality dimension, have been declining over the years, which may affect representativeness and confidence in results. The Portuguese national health examination survey INSEF (2015) achieved a participation rate of 43.9%, which is in line with participation rates from other similar health examination surveys. The objective of this article is to describe how local teams of survey personnel conducted the survey, describing strategies used to solve practical survey problems and to try to increase the participation rate.

**Methods:**

After a literature search, informal interviews were conducted with 14 public health officials from local health examination teams, regional and central authorities. Forty-one of the local staff members (survey personnel) also filled in a short questionnaire anonymously. The interviews and self-administered questionnaires were analysed using mixed methods, informed by thematic analysis.

**Results:**

The local teams believed that the detailed manual, described as a “cookbook for making a health examination survey”, made it possible to maintain high scientific standards while allowing for improvising solutions to problems in the local context. The quality of the manual, supported by a series of training workshops with the central research and support team, gave the teams the confidence and knowledge to implement local solutions. Motivation and cohesion within the local teams were among the goals of the training process.

Local teams felt empowered by being given large responsibilities and worked hard to incite people to attend the examination through a close and persuasive approach.

Local teams praised their INSA contacts for being available for assistance throughout the survey, and said they were inspired to try harder to reach participants to please their contacts for interpersonal reasons.

**Conclusions:**

The theory of organizational improvisation or *bricolage*, which means using limited resources to solve problems, was useful to discuss and understand what took place during INSEF.

A detailed manual covering standard procedures, continuous monitoring of the data collection and face-to-face workshops, including role-play, were vital to assure high scientific standards and high participation rates in this health examination survey. Close contacts between the central team and local focal points in all regions and all survey sites were key to accommodating unexpected challenges and innovative solutions.

**Supplementary Information:**

The online version contains supplementary material available at 10.1186/s13690-022-00939-7.

## Background

Information about the health of the population is a prerequisite for a national health information system (HIS). Health information and health information systems are used for policy making, to plan and implement interventions, and to monitor progress towards international and national targets [1]. Population-based health surveys are important tools for planning and monitoring [2], though non-response patterns can be problematic [3]. In general, participation rates in similar surveys have been falling over the years [3–7]. This is a complex issue, the reasons may vary between surveys, including non-suitability of time or place, illness, working hours or research fatigue, [8] but the same pattern is seen in many parts of the world [4, 6]. This article will describe how in this particular survey, many strategies were used by the local and central teams in an attempt to increase participation rates locally, and also to solve different problems that arose during the survey period.

The first Portuguese National Health Examination Survey (INSEF 2015) [9] was organised by the National Institute of Health Doutor Ricardo Jorge (INSA) in collaboration with the Norwegian Institute of Public Health (NIPH), the five Regional Health Administrations and the Regional Health Secretariats of the Autonomous Regions of the Azores and Madeira. It was financed as a project by the EEA Grants and the Portuguese Government. The survey was based on the methodology elaborated by the European Health Examination Survey network (EHES) [10–14].

The EHES methodology recommends a series of actions to increase participation rates [12]. The recommendations include informing national and local health authorities and health professionals in advance, enclosing an information leaflet with the survey invitation, having a mass media strategy, having a web page, being flexible about appointment times and re-invitations, as well as a specific method of record-keeping on eligibility and participation status for each individual invited to participate in the survey. All these recommendations were described in the Scientific Protocol [15] and followed during the project period [2, 9, 12]. Other important strategies implemented to increase participation were regular reminders [16], strict fieldwork monitoring [17], and feedback on examination and blood analysis results as an incentive [18].

The INSEF survey invited all participants to attend a simple health examination at their local health centre. These locations were chosen to ensure that people would know where to go and to ensure acceptable travel distances, as well as to inspire confidence in the participants. In some cases, examinations were carried out by staff working in that health centre or region, while in other cases staff was hired for the study. The survey had three components: measurement of height, weight, hip and waist circumference and blood pressure; collection of a blood sample for measuring total cholesterol, low density lipoprotein (LDL), high density lipoprotein (HDL), triglycerides and glycosylated haemoglobin (HbA1c); as well as a computer-assisted personal interview about socio-demography, health state, health determinants and access to care.

The INSEF target population was non-institutionalized individuals aged 25 to 74, living in Portugal for more than 12 months at the time of the survey and able to follow the interview in Portuguese. The INSEF sample was selected using a multi-stage probability sampling design in order to be representative at national level and for each of the 7 health administrative regions of Portugal. In order to estimate an expected prevalence of 50% with an absolute precision of 5% for a 95% confidence interval considering a design effect of 1.5 [9], the minimum target sample size for each region was set at 600 individuals resulting in a total of 4200 persons at the national level. Adjusted weights for non-participants were used to calibrate the sample according to the Portuguese population in 2014 [9].

The fieldwork was carried out between February and December 2015 at the 49 primary sampling units by local teams trained by the central team from INSA. Each fieldwork team consisted of two nurses, one laboratory technician (occasionally another nurse with special training) and a technical assistant, a total of 117 professionals [2, 9].

To reach the targets, 12,289 individuals were selected randomly from the national register of users of the universal national health system. This register covers all users, including migrants and temporary residents, who were considered eligible if they had been living in Portugal for more that 12 months and could communicate in Portuguese. It was possible to contact and confirm the eligibility criteria of 7784 persons. Five thousand six hundred eighty individuals were scheduled to participate, and for 4911 participants the physical examination, blood collection and interview were fully implemented [2], meaning that the national target of 4200 persons was surpassed.

The questions asked and the anthropometric, blood pressure and biochemical measurements taken were based on the recommendations from EHES [11, 12, 19], the European Health Interview Surveys (EHIS) [20] and earlier Portuguese National Health Interview Surveys [21]. The data collection was also informed by the needs for health information expressed by representatives of the health regions as part of an expert group gathered by the INSEF project.

Participation rates in health surveys have been declining over the years [4, 6, 22, 23]. This is unfortunate, as this may decrease representativeness of survey results [24]. In the EHES pilot study, participation rates varied substantially between the countries (25-63%), but for most countries, rates were below 50% [11]. The Portuguese branch of the EHES pilot study, which was carried out in Algarve in 2010, had a participation rate of 37% [25, 26]. The 2015 Portuguese Health Examination Survey INSEF achieved a participation rate of 43.9% [9]. Since INSEF was the first Portuguese national health examination survey [9], carried out by teams that had no experience of this kind, in a population with the highest illiteracy rates in Europe [27] and at least one fifth of the population presently not living in Portugal [28, 29] this may be considered quite good.

The aims of this study were to find out how local teams worked, both internally and together with the central INSA team, in the Portuguese health examination survey INSEF, and what the local teams did to improve local participation rates.

## Methods

From the beginning, a mixed methods approach, combining quantitative data from a survey directed towards the fieldwork personnel with qualitative data from the same survey and from interviews with key informants was planned. Using thematic analysis with an approach based on grounded theory [30] would allow us to gain an understanding of some underlying patterns in the practices of the local health examination teams, and as our research progressed, the rich qualitative data were allowed to be dominant in our approach. To the best of our knowledge, no studies have been published using mixed methods to discuss techniques used to address the increasing non-response rates in health surveys. This methodology would allow us to learn directly from the fieldwork personnel, while keeping in mind that some data would only be available in an aggregated form – thus, providing complementary data [31].

A literature search in the form of a scoping review was conducted (see Additional file 1 for search terms) for peer-reviewed literature about participation rates in health surveys and strategies for increasing participation rates. A librarian from the Norwegian Institute of Public Health assisted this literature search. Further search for more literature was performed using both the snowball method [32], following the references in the literature found in the primary search, and the database from the NoPaHES (Non-participation in Health Examination Surveys) project, which contains literature on non-response and ways to handle it with statistical methods [33].

### Quantitative data collection and analysis

An online questionnaire (Additional file 2) was constructed, and the link was distributed to all seven regional heads of the INSEF survey in September 2016 in connection with a project meeting at INSA for further dissemination. All 117 members of local survey teams plus the seven regional heads were assumed to have received the questionnaire, which constituted a total of 124 individuals. Forty-one filled-in questionnaires were returned by October 11th, resulting in a response rate of 33%. We chose to ask these questions in an anonymous questionnaire instead of face to face to make it easier for survey personnel to speak openly about their organisations and colleagues, and so they would have more time to think about each question.

The respondents were high-level coordinators (17%), laboratory workers (17%) and local coordinators, assistants, nursing students and nurses (the remainder). No further information was collected about these respondents to ensure they would feel safe to disclose all kinds of information, without feeling they could be identified by INSA staff.

The questionnaire had 16 questions: 8 questions had coded (numerical) answers and 8 were open questions. See Additional file 2 for the entire questionnaire.

### Qualitative data collection and analysis

In October 2016, the first author conducted semi-structured interviews, based on a loose interview guide (Additional file 3) with 14 public health officials connected to INSEF, from local health examination teams and regional and central authorities. The interviews were conducted during an INSEF meeting in Lisbon and at the central offices of INSA. The number of interviews required was not set from the start and was planned to depend on the available time. Quite early in the interview process, the newer interviewees started repeating what earlier interviewees had said, which indicated that we might be approaching data saturation [34]. Nevertheless, another few interviews were conducted, bringing the number of interviews up to 14. The interviewees were chosen at random (the first person who walks through a door), but a few people refused because they did not speak English. The results were transcribed by the first author and discussed with the second author, who was also present during the open line-by-line coding of both the interview transcripts and the free-text replies from the 41 anonymous questionnaires. What was of interest was the perspectives of the people working on INSEF, encoded using thematic analysis [30]. The free-text replies repeated many of the topics from the oral interviews, which also indicated data saturation [34].

The codes were compared and clustered into sub-categories, and from these the final themes were constructed. These 55 survey personnel, who consisted of 14 interview objects and 41 questionnaire respondents, were the main informants in this study. The majority of the interview objects were women, under the age of 50. There was no identifying information about the survey personnel who filled in the questionnaire, but we have allowed them a voice by using their direct words to illustrate the analysis.

In addition, the following materials, not originally produced for this study, were obtained and coded, and used as supporting information:

- The transcript from one of the survey personnel focus groups conducted locally by the second author [35], with 17 participants from six of the seven Portuguese health regions, conducted with the local health examination teams, translated to English to ensure that the first author would understand;
- Three PowerPoint presentations made by regional heads of the survey, including SWOT analyses;
- Notes from workshops held during the preparatory and training phases of INSEF on the physical examination, the blood sampling and analysis, and the questionnaire interviews

The coding and the topics identified in the thematic analysis were discussed between the first two authors, and the results were shared between all the authors for comments and revisions. Other materials (Additional file 4) were also consulted but were not coded and did not form part of the thematic analysis.

### Mixed method analysis

The materials above, the interviews and the questionnaire texts, were analysed using thematic analysis, informed by grounded theory [30], and the results were combined with the simple qualitative data from the questionnaires as a basis for a mixed method analysis [36], seen in light of the relevant literature. Mixed methods have been a fruitful way of regarding the available data and has made it easier to see more dimensions of the research question than any single approach. We have used a pragmatic approach [31], where the qualitative data have been allowed to dominate the quantitative data because the interviews and free texts were so much richer than the simple questionnaire data.

## Results

Results from this study are presented in separate sections, reflecting the mixed methodology used.

### Quantitative data from questionnaires

A survey question on whether the survey personnel respondents had been happy or unhappy to take part in the survey, was met by overwhelmingly positive response. 95% of the fieldwork team members who took part in the survey were happy that they had participated in INSEF, with 83% reporting being very happy to have participated. 2% were neither happy nor unhappy, and 3% were unhappy. None reported being very unhappy that they participated.

**Fig. 1:**
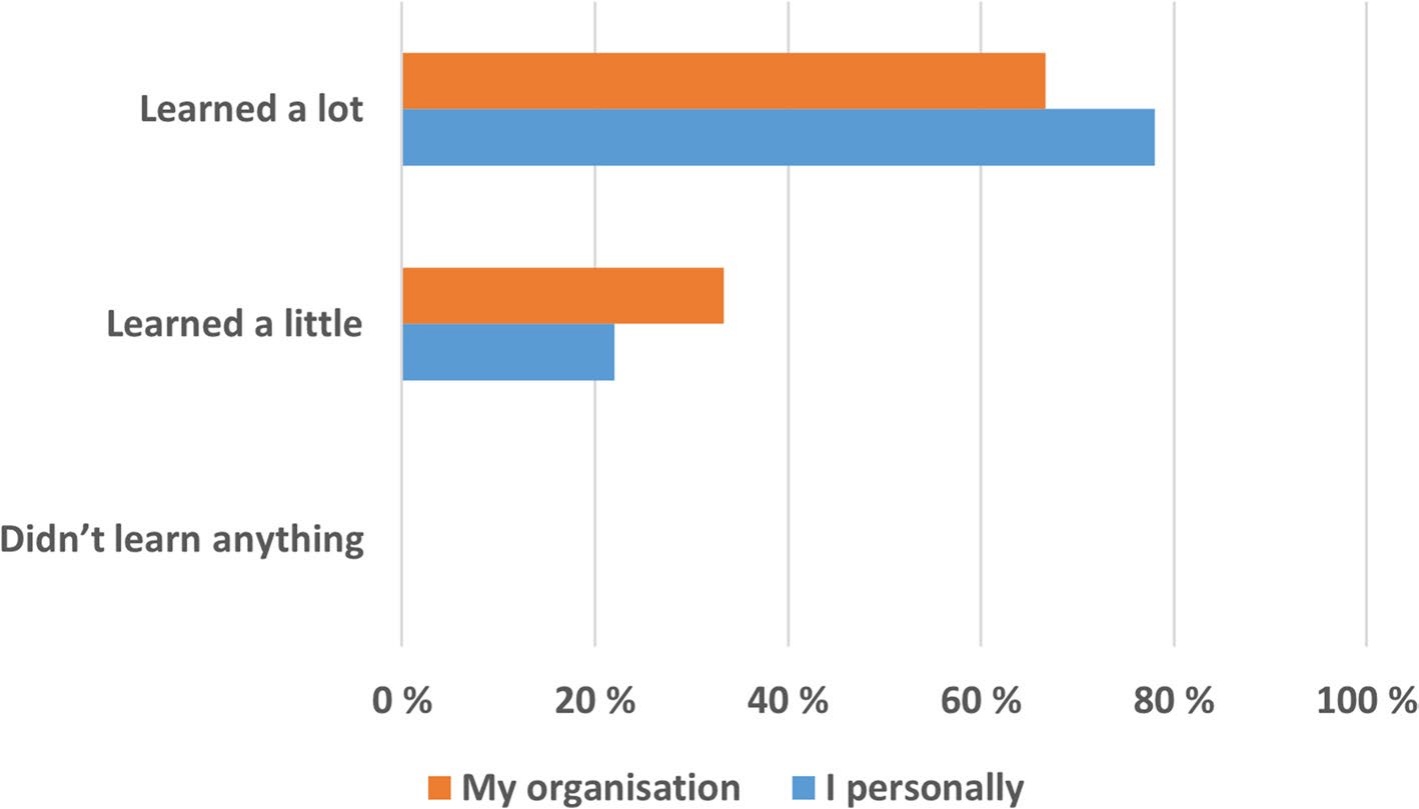
Did the survey personnel and their organisations learn anything by taking part in INSEF? (per cent)

Figure 1 shows that both the survey participants and their organizations learned by taking part in the survey. The majority felt that both they personally and their organisations had learned a lot, and no participants reported that they/their organisations didn’t learn anything.

Another survey question, “Would you (personally) do another round of INSEF in a few years?” demonstrated overwhelming support, in that 55% of the survey personnel respondents would absolutely participate in another round of INSEF if given the chance, and 29% would probably participate, while only 12% were unsure. No respondents stated that they would absolutely not participate in another round of INSEF.

The survey also demonstrated a wide distribution on how easy or hard the survey personnel respondents had found it to get their organisations to cooperate regarding INSEF: 11.9% found it very hard, 14.3% hard, 28.6% neither hard nor easy, 19.0% easy, and 26.2% found it very easy to get their organisation to cooperate regarding INSEF.

In addition to the questions described above, there was also a multiple choice-question (up to 3 possible answers) about the local teams’ the most difficult issues with running INSEF. The replies chosen by most survey personnel respondents were *Getting personnel* (50%) and *Getting time to do it (45%)*. The other response alternatives, *Getting local permission*, *Getting local funding*, *Cooperating with health centres*, *Practical problems regarding examination* and *Cooperating with labs* were selected by between 7 and 20% of the survey personnel respondents. No survey respondents selected *Cooperating with INSA* or *Training personnel*.

Finally, 83% of the survey personnel respondents thought it would be useful to have another INSEF in a few years, while 17% thought it would be somewhat useful. None of the respondents replied that it would not be useful.

### Qualitative data from the questionnaires

One of the main goals of INSEF was achieving a high participation rate to ensure representative results describing the Portuguese population [9].

**Fig. 2:**
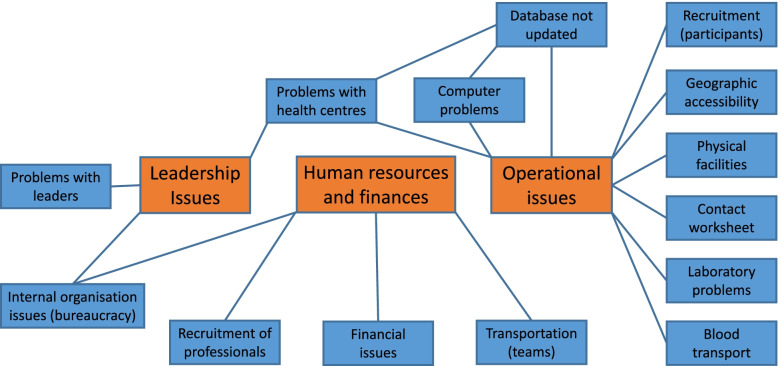
Codes and Themes used in the thematic analysis

Table 1, below, consists of a list of *codes* chosen during the thematic analysis [30] as issues or problems that made it hard to achieve both a sufficient number of participants for INSEF and to do the day-to-day work of getting the measurements, samples and filled-in questionnaires from each participant. The section below the table explains what the codes mean. These codes describe issues that were recurrent in the interviews, the free text from the questionnaires and indeed, all the documentation that was consulted while writing this article. The codes have been sorted into overarching themes. In Fig. 2, the codes are sorted using Thematic Analysis. The diagram shows how the codes are connected to the overarching themes. See also the code list, Additional file 5.

**Table 1:** Issues that the survey personnel respondents thought made it harder to perform the INSEF survey

Themes	Codes
Leadership issues	Problems with leaders
	Internal organisation issues (bureaucracy)
	Problems with health centres
Human resources and financial issues	Recruitment of professionals
	Financial issues
	Internal organisation issues (bureaucracy)
	Transportation (teams)
Operational issues	Contact worksheets
	(Local) database not updated
	Computer problems
	Laboratory problems
	Geographic accessibility
	Problems with health centres
	Recruitment (participants)
	Physical facilities
	Transportation (participants)

### Explanation of the codes

- *Problems with leaders* (and their (missing) involvement)
  Many informants expressed the opinion that they achieved what they did in spite of, not because of, their bosses. This was particularly common among nurses who were employed by the regions. “Managers uninterested in project” and “bureaucracy among high-placed managers” were replies to informant survey question about what made it hard to get their organisation to cooperate.
- *Internal organisational issues* (bureaucracy)
  Among the reported issues was changing decision-makers locally during the project period, as well as a lack of knowledge about the survey in higher managers and late payment to nurses and technicians.
- *Problems with health centres*
  Not all health centres collaborated seamlessly with the local teams, and there were complaints about locked doors, receptionists who sent participants home claiming there was no health examination survey taking place, and surly security guards.
- *Recruitment of professionals*
  It was difficult to get enough nurses and technicians to conduct the study among regular health centre staff, and some newly educated nurses were recruited just for the survey. All these professionals had to be available on fairly short notice to travel from health centre to health centre within their regions. In some locations, employees had to perform the survey in addition to their normal tasks.
- *Financial issues*
  Taking part in the project required allotment of scarce resources locally, and middle management, who were given the responsibility to carry out the survey, did not always get the extra resources needed to do so, including overtime payment for the nurses
- *Internal organisation issues* (bureaucracy).
  Negative internal collaboration within team performing the fieldwork.
- *Transportation* (teams)
  Getting the teams to their temporary working places to perform the survey activities. This was a special problem on the Azores, where some of the local team members had to travel from island to island to do their jobs, but many teams on the mainland also reported that they had problems getting to the health centres.
- *Contact worksheets*
  The (paper) data collection instruments for recruitment, listing names and contact data for the people selected to take part in the survey and questions about outcome of contact attempts and eligibility were made by the central INSA team, and some local technicians found them difficult to fill out.
- *Sampling frame not updated*
  This refers to the database of health centre users which was used to sample and contact INSEF participants, which was not completely up to date with contact details for all selected individuals.
- *Computer problems*
  All the local teams were provided with laptops by INSEF, but some teams had little experience using these tools. RedCap Electronic Data Capture Software [37], which was used by the survey teams, required an internet connection for functioning, and in some locations, the internet connection was not good enough.
- *Laboratory problems*
  The blood samples were processed at local laboratories by the local teams, and there were problems getting time and space in the laboratories as well as trained lab technicians to process the blood samples.
- *Geographic accessibility* (survey participants and blood samples)
  It was at times problematic to get the participants from their homes to the health centres for examinations in areas with little public transport.
  In the survey, blood had to be kept in refrigerators or in special transport boxes and transported to laboratory hubs within a short time period to be processed.
- *Problems with health centres*
  Some health centre facilities did not have enough space for the local teams to do their jobs without disturbing the usual health centre activities, and there were health centres where the local teams were met with a lack of understanding or even hostility.
- *Recruitment of participants*
  This is a major challenge for all health examination surveys [4, 18, 24, 38]. Statements and references from the local teams about techniques and strategies they employed to recruit participants are included here.
- *Physical facilities*
  The examinations were carried out in rooms that belonged to local health centres, and not all these rooms were suited for the purpose. There were reports of lacking handwashing facilities, uncomfortable temperatures for the study participants and no place for the participants to sit while their blood was collected.
- Transportation of participants

As one can see from Fig. 2, many issues that were considered problems in reaching the goal of INSEF were identified. There are fewer items on the list of issues that were identified as positive for recruitment and performance of the INSEF survey, because so many of the fieldwork team members who filled in the questionnaires and took part in the focus group and the interviews pointed to the same things.

Issues that made it easier to perform the INSEF survey, identified by several members from the local survey teams:

- *Training*
  The central team from INSA invited the local teams of survey personnel to regional centres for training in how to recruit participants, perform the measurements, take blood samples and fill in the online CATI questionnaires, and then role-played the whole scenario.
- *The protocol*
  Several fieldwork team members, and some members of the central INSA team, called the study protocol, based on the EHES manual [14] “the cookbook”, which emphasizes that the protocol was thought to be a clear description of exactly what was supposed to happen at each step of the health examination survey.
- *Internal collaboration within team* of survey personnel
  During the interviews, particularly the youngest fieldwork team members talked about how nice it was to work in a team of colleagues and about how much they had learned from working in this way. One elderly team member had such a good time that they would like to come out of retirement, if necessary, to participate in any future INSEF.
- *Positive communication with INSA* (the central team)
  Both direct communication, in groups or one-to-one, and indirect communication, such as the newsletters and Field barometers were seen in a very positive light.
- *Informal strategies to solve problems*
  How the local teams felt empowered and proud of their ingenious ways of solving any problems that occurred, while remaining faithful to the protocol.

Focusing on the last two points, the following subsection of the results details the analysis, with particular focus on the local teams’ informal strategies to solve problems found in the free text answers from the survey personnel respondents – grouped according to these issues.

#### Positive communication with INSA (the central team)

The free text replies from the workers on the local teams were all positive, as seen from the following examples. The number of replies praising the work of the central INSA team was, in fact, quite overwhelming.

E1: ‘Yes, they (INSA) were spectacular in trying to solve problems and doubts that the field teams had, as well being ready to support and assist any difficulties that they experienced.’

E2: ‘The INSEF team at INSA was tireless and always available. I think it would be (good) to keep the training model and also the presence of INSA team on the first day of the project at each site. The previous visit of INSA staff to health facilities is also essential, in the project dissemination perspective and to see the facilities, in order to verify their suitability.’

E3: ‘The role (INSA played as) as facilitator during this INSEF was very good. They should do in same way in the future.’

E4: ‘… we had the INSA Team who helped us with the head of the institution.’

The good work of the INSA team was also mentioned in the focus group transcript, but since two INSA representatives led the group, this was not surprising. There were no negative statements about the INSA team – see also the Quantitative results, where there were no survey personnel respondents who thought it was difficult to collaborate with INSA. In the informal interviews, as well as in the focus group transcripts, there was also a lot of praise for the manual (called “the cookbook” by several respondents), which was very thorough and had solutions for many kind of foreseen problems.

#### Informal strategies to solve problems – direct quotations from the questionnaires

E5: ‘When users were illiterate (could not read) I tried to simplify/explain the best possible response options. Invested more time in these situations.’

E6: ‘The flow chamber* was not always available, which forced us to use it only when it was available and much time was needed for working outside ordinary working hours and / or working late. (*refers to lab equipment for aliquoting blood samples)’

E7: ‘The failure of the computer system of our service. It was resolved with the completion of all manual samples and delivery of results on paper.’

E8: ‘Health centres do not have a specific room for this service (reception room to receive samples), so we improvised a blood collection room.’

E9: ‘We performed the initial training without authorization from the leaders, which was only given later.’ (refers to top-level managers, not direct superiors)

E10: ‘Difficulty of access (to health centre), but we carried the person*’

(*the local team physically carried the respondent up the stairs to the examination room at the health centre)

E11: ‘Transportation difficulties in moving staff and blood samples. We used the private car of a member of staff.’

E12: ‘Sometimes there was a lack of communication and we solved that with a meeting.’

E13: ‘We did not have appropriate rooms, but with the cooperation of all, we decided the best way and it went very well.’

Other informal strategies that were mentioned in the interviews were using private cars to transport survey participants (E14) and phoning participants at weekends and in the evening (E15), both invitations and reminders were conveyed in this manner. Several examination sites decided to expand opening hours to reach working people (E16). In a case where there was no suitable chair for taking blood samples, one nurse obtained the key to a physician’s office currently not in use and borrowed a chair (E17). One site found they needed to overbook to get enough participants to attend, and started serving coffee and tea when the waiting room was full to placate survey participants who were waiting to answer the questionnaire after the physical measurements had been taken (E18).

These and other informal strategies were employed by the local teams to solve the problems that were not foreseen by the central INSA team, and therefore were not featured in “the cookbook”. Several of the informal interviews had this as a central topic, and the first author became interested in the culture that allowed such “outside the box” thinking and problem solving [39], even by young employees such as the newly graduated nurses who constituted a large number of elements of the local teams.

**Fig. 3:**
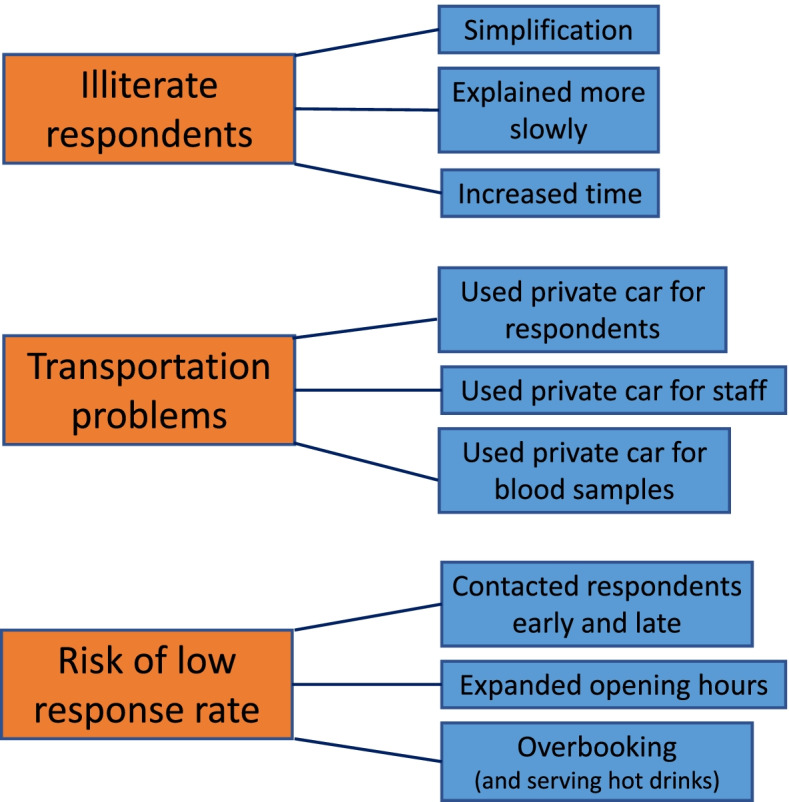
Problems and informal solutions identified by the survey personnel respondents

Figure 3 (below) diagrammatically demonstrates some of the problems and the informal solutions that members of the local teams reported. The problems are shown in the orange boxes, while the blue boxes describe some of the methods that were used to fulfil the goals of the INSEF survey.

### Interpretation

Local teams were highly motivated to take part in the INSEF survey, this is demonstrated by both the quantitative and the qualitative material. According to the interviews, they felt empowered by being given large responsibilities and worked hard to incite the invited people to attend the examination, including telephoning them outside work hours and using private cars to get people with reduced mobility or transport problems to the examination sites. One of the authors also reported that many local team members liked having a certificate of training, signed by high-level officials at INSA, to put in their Curriculum Vitae.

Both the local teams and their superiors believed that the manual, described informally at project meetings and interviews as a “cookbook for making a health examination survey”, made it possible to maintain high scientific standards while at the same time improvising local problem solving, which was suitable in the local context. The quality of the manual, supported by a series of training workshops, where the local teams met people from the INSA research and support team and role-played different scenarios, gave the teams the confidence and knowledge to implement local solutions.

After the training sessions in each region a pilot day, observed by the trainers, was organised, followed by a meeting to evaluate the experience and identify difficulties, doubts, suggestions, etc. For each region, a short report was written, listing issues for improvement. These regional reports, mostly available as PowerPoint files, further demonstrate the ingenuity and inventiveness of the local teams. These reports show how motivated the local teams were to participate in the survey.

Local teams praised their INSA contacts, and said they were inspired to try harder to reach participants to please their contacts for interpersonal reasons. This is also supported by the questionnaire results, where there weren’t any survey personnel respondents who reported that *Cooperating with INSA* or *Training personnel* had been among the three most difficult things about participating in INSEF.

These results can be explained by the close and demanding approach that was chosen throughout the entire process of implementing INSEF, a continuous improvement process. This is also documented by Kislaya et al. [2, 40].

### Other actions undertaken to improve participation rates

To achieve acceptable participation rates in a health examination survey, creating increased public awareness about the upcoming and ongoing survey via a public relation campaign is likely to be useful [13, 41], and several such campaigns were planned and implemented, both nationally and regionally, in close collaboration with the services of the seven health administrations [16].

The INSEF survey included only the non-institutionalised population [9], meaning that some of the oldest persons were not sampled, since many live in nursing homes. Even for people living at home, it may have been difficult to attend the health examination survey because of functional limitations and transportation issues [7]. Some of the improvised solutions, such as using private cars (E10) and carrying people up the stairs (E6) to get the most infirm and people without access to transportation to attend the health examination survey, were considered helpful.

Reminders by phone, SMS or direct contact, are known to increase the participation rate in health examination surveys [16, 42], and the protocol included making 1 to 5 telephone contacts to reach the invited individuals. The local teams on their own initiative started telephoning prospective participants outside working hours (E11) and managed to reach people who were at work in the daytime. SMS contacts were also used when mobile numbers could be obtained [16].

A common reason for non-participation in health surveys is that the time or place is inconvenient or not suitable for the person [8]. Offering private transportation (E10) and expanded opening hours (E12) were attempts to alleviate this in INSEF, as was offering the participants the laboratory results from their blood sampling procedures.

## Discussion

Health surveys are needed to obtain information about health status and health-related behaviours in the population. To obtain the best possible results, high participation rates and representative participation are required [4, 5]. In this survey, it was clear from the beginning that monetary incentives, which have not be shown to be effective [4], would not be used [9].

In comparable European countries, participation rates in health examination surveys have fallen over the years [4, 8, 18]. The promoters of the INSEF survey were aware of this, and tried to use many different techniques to increase participation rates [13]. Many of these techniques were detailed in the study protocol and the materials from the INSA-NIPH meetings, such as dissemination methods at national and local level, flexibility in the data collection period and close monitoring of participation rates, so issues could be identified quickly and changes implemented [9, 35, 40, 43]. The weekly Fieldwork Barometers were both a tool for surveillance of the ongoing survey and for encouraging the local survey teams.

The European Health Examination Survey handbook recommends a strong leadership model, with a Steering Committee, a Project Manager and a Core group supporting the Survey Teams [13]. The questionnaires filled in by the Survey Teams were full of praise for the Core group, also described here as the central team. Several members of the Core group expressed satisfaction with the role played by the consultant from the Norwegian Institute of Public Health, whose role as both an insider and an outsider made it possible to observe the health survey in a unique way.

The central EHES model [13] was used, but the questionnaires and interviews identified that though the survey teams referred to and tried very hard to follow the protocol (“the cookbook”), many decisions were made informally and at a lower level than the protocol or the EHES handbook recommend. Local teams used their best judgement to adapt to local circumstances and unexpected situations and suggested new solutions after conferring with the guidelines and in close communication with the central team at INSA.

Examples: ‘We improvised a blood collection room’ (E8), ‘We performed the initial training without authorization from the leaders, which was only given later’ (E9).

Pina e Cunha describes Portuguese management culture as feminine and collectivist [39]. By that, he means that relationships and cooperation are highly valued, and also describes a management practice where rules imposed from above and what is needed in the local context are seamlessly merged. One of Pina e Cunha’s interview objects puts it like this: “When there’s a Portuguese there’s no problem. Everything gets solved. Things never stop.” [39]. Kamoche et al. underline that researchers need to pay more attention to “voluntarist, emergent and creative behaviors” [44], and we believe that is what the problem-solving of the local teams represents.

There were situations that had not been anticipated at INSA’s level, nor reported there, but solved locally, in the spirit of the protocol and the extensive training. Local teams put together available resources and local knowledge with the written plans using a great deal of ingenuity, being highly motivated to succeed and be seen as partners in a scientific study.

Terms used for this kind of innovative improvisation within organisations are *bricolage* and *organizational improvisation* [45]. An additional literature search outside the health field led to a number of authors who describe this, mostly within the fields of organizational theory and entrepreneurship.

Pina e Cunha defines bricolage as using whatever resources people have at hand to reach their goals [46]. Bacq et al. [47] describe it as “the creation of something new (…) through recombination and transformation of existing resources.” The nurse who made off with a chair from another office, since there was a need for a chair for blood sample taking (E16), was practicing bricolage, and so were the local teams who telephoned prospective participants at weekends to reach people outside their working hours.

Bacq et al. underline that bricolage, as a process, is a way for people with few resources to use what they have to solve the problems they need to solve, and that social resources and networks are important in this kind of improvisation [47]. This seems to be what was going on for the local teams, and the ways they worked together to ensure as many INSEF participants as possible. The weekly newsletters, referred to as “Barometers”, provided by the central INSA team also helped to foster good collaboration within the teams (E5, E8, E9), who in good-natured competition tried to be at least as good as the other teams (interview). The continuous monitoring of local results was also important in order to give feedback on local issues, such as potential misunderstandings regarding protocol, as quickly as possible [2, 40].

It was important that the health examinations were done at the health centres people already went to, and that the examinations were performed by locals, including nursing graduates from the local area [35]. This made local, organizational adaptation [44, 46, 48] possible, which seems to be one of the keys to the successful outcome of INSEF. The local teams knew the local context, partially since some of them already worked in the health centres where the examinations took place, and they used local knowledge and information about local situations to persuade people to come for a health examination (information from interviews and focus group).

Personal contacts seem to have been important for the local teams to feel included and safe enough to suggest local solutions. Though the INSA core group were the public health experts, these nurses were the experts on the local population (from interview). The way they followed the protocol as closely as possible, and made small adaptations when local conditions, such as lack of transportation for blood samples and examination teams (E7) made it necessary, exemplifies bricolage [46, 47, 49].

We believe that this creative use of scant resources was one of the key reasons that the INSEF survey, despite all the problems mentioned, succeeded in getting a relatively large number of participants.

### Strengths and limitations

This study is limited by the single location studied. The results from Portugal may not be transferrable to other countries.

The study deals only with health surveys and may not be relevant for other kinds of surveys.

We do not have any sociographic information about the survey personnel who participated in the survey, and the participation rate was quite low, so we cannot know whether the survey was representative.

The interviewees were randomly chosen among the people physically present but may not be representative for all the survey teams, since people who did not speak English at all had to be excluded.

The last fieldwork was carried out in December 2015, but the questionnaires were sent out and the interviews were carried out in September and October 2016. This means that recall bias may be an issue, but we hope that in performing the survey and the interviews around the same time as a well-attended meeting for the focal points from Portugal’s health regions where INSEF was the main topic (September 2016), we managed to remind the survey personnel questionnaire respondents and interviewees about the project.

The study has the following strengths:

- The first author’s outsider perspective made it easier for the interviewees to talk freely, without fearing negative responses from the central INSEF team after criticism.
- The direct use of the survey personnel respondents’ own words, ensuring that they were truly heard.
- The use of an online questionnaire, ensuring full anonymity for the survey personnel respondents, who could be truthful without fearing reprisals from their superiors or the INSA team.

## Conclusion

The first Portuguese health examination survey, INSEF, managed to get acceptable response rates by functioning according to the central INSA team’s slogan: “Train, evaluate, improve!”. INSEF had an extensive manual covering standard procedures and a number of face-to-face workshops, so the local teams got to know the central team. INSEF used local teams with good contacts, and allowed the local teams to improvise, in the spirit of the manual, when lack of resources or local support made it necessary.

Informal strategies, based on solid knowledge in the form of the manual, were important to solve problems during data collection, and this can be an important teaching for other countries wishing to increase participation rates in health examination surveys. The participatory methodology used in INSEF had the flexibility to listen to people, to train and evolve, and was a good way to obtain the motivated participation of the local health teams, which is essential to get enough participants.

## Supplementary Information

**Additional file 1:** Search terms.

**Additional file 2:** Questionnaire for local survey team members.

**Additional file 3:** Informal interview guide.

**Additional file 4:** Supplementary materials.

**Additional file 5:** Code list used in Thematic Analysis.

## Data Availability

Data used in this study are available upon request from the first author at the Norwegian Institute of Public Health (NIPH) or from INSA after permission by INSEF’s Evaluation Board.

## Abbreviations

EEA: European Economic Area
EHES: European Health Examination Survey
INSA: Instituto Nacional de Saúde Doutor Ricardo Jorge (the Portuguese national public health Institute)
INSEF: Inquérito Nacional de Saúde com Exame Físico (the first Portuguese national health examination survey)
NIPH: Norwegian Institute of Public Health

## References

[1] Verschuuren M, van Bolhuis A, Rosenkötter N, Tijhuis M, van Oers H (2017). Towards an overarching European health information system. Eur J Pub Health.

[2] Kislaya I, Santos AJ, Lyshol H, Antunes L, Barreto M, Gaio V, Gil AP, Namorado S, Dias CM, Tolonen H (2020). Collecting valid and reliable data: fieldwork monitoring strategies in a health examination survey. Port J Public Health.

[3] Hohwü L, Lyshol H, Gissler M, Jonsson SH, Petzold M, Obel C (2013). Web-based versus traditional paper questionnaires: a mixed-mode survey with a Nordic perspective. J Med Internet Res.

[4] Tolonen H, Ahonen S, Jentoft S, Kuulasmaa K, Heldal J, Project EHEP (2015). Differences in participation rates and lessons learned about recruitment of participants–the European Health Examination Survey Pilot Project. Scand J Public Health.

[5] Conway DI, McMahon AD, Smith K, Taylor JC, McKinney PA (2008). Socioeconomic factors influence selection and participation in a population-based case–control study of head and neck cancer in Scotland. J Clin Epidemiol.

[6] de Leeuw E, Hox J, Luiten A (2018). International nonresponse trends across countries and years: an analysis of 36 years of Labour Force Survey data. Survey methods: insights from the field.

[7] Gaertner B, Seitz I, Fuchs J, Busch MA, Holzhausen M, Martus P, Scheidt-Nave C (2016). Baseline participation in a health examination survey of the population 65 years and older: who is missed and why?. BMC Geriatr.

[8] Tolonen H, Lundqvist A, Jääskeläinen T, Koskinen S, Koponen P (2017). Reasons for non-participation and ways to enhance participation in health examination surveys—the health 2011 survey. Eur J Public Health.

[9] Nunes B, Barreto M, Gil AP, Kislaya I, Namorado S, Antunes L, Gaio V, Santos AJ, Rodrigues AP, Santos J (2019). The first Portuguese National Health Examination Survey (2015): design, planning and implementation. J Public Health.

[10] Kuulasmaa K, Tolonen H, Koponen P, Kilpeläinen K, Avdicová M, Broda G, Calleja N, Dias C, Gösswald A, Kubinova R (2012). An overview of the European health examination survey pilot joint action. Arch Public Health.

[11] Tolonen H, Koponen P, Mindell J, Männistö S, Kuulasmaa K (2014). European health examination survey—towards a sustainable monitoring system. Eur J Public Health.

[12] Tolonen H, Koponen P, Aromaa A, Conti S, Graff-Iversen S, Grøtvedt L, Kanieff M, Mindell J (2008). Recommendations for organizing a standardized European health examination survey. B22/2008.

[13] Tolonen H (2016). EHES manual. Part A. Planning and preparation of the survey.

[14] Tolonen H (2016). EHES manual. Part B. Fieldwork procedures.

[15] INSEF project team (2014). INSEF 2013-2016 Scientific protocol.

[16] Sääksjärvi K, Koponen P, Tolonen H, Koskinen S, Lundqvist A, Kontto J, Borodulin K (2018). How to increase participation in health examination surveys? Findings from the FinHealth 2017 survey. Eur J Pub Health.

[17] Meitinger K, Ackermann-Piek D, Blohm M, Edwards B, Gummer T, Silber H (2020). Fieldwork monitoring strategies for interviewer-administered surveys. Survey methods: insights from the field.

[18] Mindell JS, Giampaoli S, Goesswald A, Kamtsiuris P, Mann C, Männistö S, Morgan K, Shelton NJ, Verschuren WM, Tolonen H (2015). Sample selection, recruitment and participation rates in health examination surveys in Europe–experience from seven national surveys. BMC Med Res Methodol.

[19] Tolonen H, Koponen P, Capkova N, Giampaoli S, Mindell J, Paalanen L, Ruiz-Castell M, Trichopoulou A, Kuulasmaa K (2018). European health examination surveys–a tool for collecting objective information about the health of the population. J Arch Public Health.

[20] Hintzpeter B, Finger JD, Allen J, Kuhnert R (2019). European health interview survey (EHIS) 2–background and study methodology. J Health Monit.

[21] Alves J, Kunst AE, Perelman J (2015). Evolution of socioeconomic inequalities in smoking: results from the Portuguese national health interview surveys. BMC Public Health.

[22] De Heer W (1999). International response trends: results of an international survey. J Off Stat.

[23] Tolonen H, Helakorpi S, Talala K, Helasoja V, Martelin T, Prättälä R (2006). 25-year trends and socio-demographic differences in response rates: Finnish adult health behaviour survey. Eur J Epidemiol.

[24] Drivsholm T, Eplov LF, Davidsen M, Jørgensen T, Ibsen H, Hollnagel H, Borch-Johnsen K (2006). Representativeness in population-based studies: a detailed description of non-response in a Danish cohort study. Scand J Public Health.

[25] Tolonen H (2013). Proceedings of the European Health Examination Survey Conference-monitoring the health of Europeans. Monitoring the health of Europeans: 2013.

[26] Department of Epidemiology I. EHES joint action progress report January 2010-20 June 2010 - preliminary results of the pilot phase. Lisbon; 2010. p. 31.

[27] Roser M, Ortiz-Ospina E (2016). Literacy. 2016th edn.

[28] At least one-fifth of people from these countries lives abroad. https://www.weforum.org/agenda/2016/12/fifth-of-people-from-these-countries-live-abroad/#:~:text=A%20recent%20study%20by%20the,also%20left%20their%20native%20home. Accessed 2 Aug 2022.

[29] United Nations. International migration report 2015: highlights. Edited by Department of Economic and Social Affairs PD, vol. ST/ESA/SER.A/375. New York: United Nations; 2016.

[30] Braun V, Clarke V (2013). Successful qualitative research: a practical guide for beginners.

[31] Halcomb EJ, Hickman L (2015). Mixed methods research. Faculty of Science, Medicine and Health - Papers: part A. edn.

[32] Perez-Bret E, Altisent R, Rocafort J (2016). Definition of compassion in healthcare: a systematic literature review. Int J Palliat Nurs.

[33] Literature on non-response and ways to handle it with statistical methods. http://www.ehes.info/nopahes/index.html.

[34] Guest G, Bunce A, Johnson L (2006). How many interviews are enough? An experiment with data saturation and variability. Field Methods.

[35] Gil AP, Santos A, Santos J, Kislaya I, Rodrigues A, NamoradoV S, et al. Population’s adherence to the Portuguese health examination survey: the perspective of fieldwork teamsAna Paula Gil. Eur J Pub Health. 2015;25(suppl_3):417.

[36] Hesse-Biber SN (2010). Mixed methods research: merging theory with practice.

[37] Wright A (2016). REDCap: a tool for the electronic capture of research data. J Electron Resour Med Libr.

[38] Dillman DA, Phelps G, Tortora R, Swift K, Kohrell J, Berck J, Messer BL (2009). Response rate and measurement differences in mixed-mode surveys using mail, telephone, interactive voice response (IVR) and the internet. Soc Sci Res.

[39] Cunha MP (2005). Adopting or adapting? The tension between local and international mindsets in Portuguese management. J World Bus.

[40] Kislaya I, Rodrigues AP, Santos J, Gaio V, Gil AP, Santos AJ, Namorado S, Barreto M, Lyshol H, Nunes B (2015). Portuguese National Health Examination Survey: lessons from data collection monitoring. 8th European public health conference: 2015.

[41] Gößwald A, Lange M, Dölle R, Hölling H (2013). The first wave of the German health interview and examination survey for adults (DEGS1). Bundesgesundheitsblatt.

[42] Tolonen H, Aistrich A, Borodulin K. Increasing participation rates by SMS reminders and flexible examination hours in Kuusamo health examination survey in FinlandKatja Borodulin. Eur J Pub Health. 2014;24(suppl_2). 10.1177/1403494814544403 25118200

[43] Chaudhuri S, Morash M (2019). Monitoring team interviews during fieldwork: some lessons from India. Int J Sociol.

[44] Kamoche K, Pina e Cunha M, Campos e Cunha R. Preface: improvisation in organizations. Int Stud Manag Organ. 2003;33:3–9.

[45] Senyard J, Baker T, Steffens P, Davidsson P (2014). Bricolage as a path to innovativeness for resource-constrained new firms. J Prod Innov Manag.

[46] Cunha M. Bricolage in organizations. SSRN Electron J. 2005:1–32.

[47] Bacq S, Ofstein LF, Kickul JR, Gundry LK (2015). Bricolage in social entrepreneurship: how creative resource mobilization fosters greater social impact. Int J Entrepreneurship.

[48] Cunha MP, da Cunha JV, Kamoche KN. Organizational improvisation. Int J Manag Rev. 2002;96.

[49] Desa G (2012). Resource mobilization in international social entrepreneurship: Bricolage as a mechanism of institutional transformation. Entrep Theory Pract.

