# Supplementary material for: Local problem solving in the Portuguese health examination survey: a mixed method study
Source: Arch Public Health. 2022 Aug 24;80:198. doi: 10.1186/s13690-022-00939-7 (PMC9400230; doi:10.1186/s13690-022-00939-7)
Supplement: Supplementary file 1 — Additional file 1: Search terms. [file 13690_2022_939_MOESM1_ESM.docx]

## Additional file 1: search terms

How to achieve high participation in health examinations?

| **Contact person:** | Heidi Lyshol |
| --- | --- |
| **Search:** | Nataliya Byelyey, NIPH |
|  |  |

**Database: Ovid MEDLINE(R) In-Process & Other Non-Indexed Citations, Ovid MEDLINE(R) Daily and Ovid MEDLINE(R) 1946 to Present**

**Date:** 26.06.2017

**Hits:** 567

|  |  |  |
| --- | --- | --- |
| 1 | "surveys and questionnaires"/ or health surveys/ | 432295 |
| 2 | ("health examination survey*" or HES*).tw. | 23974 |
| 3 | 1 or 2 | 455553 |
| 4 | ((high* or increas*) adj (respons* or particip*) adj2 rate?).tw. | 6211 |
| 5 | 3 and 4 | 567 |
|  | | |

**Database: PsycINFO**

**Date:** 26.06.2017

**Hits:** 207

|  |  |  |
| --- | --- | --- |
| 1 | exp SURVEYS/ | 8522 |
| 2 | (health adj2 survey?).tw. | 11228 |
| 3 | 1 or 2 | 19057 |
| 4 | ((high* or increas*) adj (respons* or particip*) adj2 rate?).tw. | 1263 |
| 5 | 3 and 4 | 207 |

**Database: Embase 1974 to 2017 July 05**

**Date:** 05.07.2017

**Hits:** 329

| 1 | exp health survey/ | 192619 | Advanced |
| --- | --- | --- | --- |
| 2 | (health adj2 survey?).tw. | 41526 | Advanced |
| 3 | 1 or 2 | 214011 | Advanced |
| 4 | ((high* or increas*) adj (respons* or particip*) adj2 rate?).tw. | 8803 | Advanced |
| 5 | 3 and 4 | 329 | Advanced |

**Database: Scorpus**

**Date:** 05.07.2017

**Hits:** 53

( TITLE-ABS-KEY ( *"health examination survey"* )  AND  TITLE-ABS-KEY ( ( *high**  OR  *increas** ) )  AND  TITLE-ABS-KEY ( ( *respons**  OR  *particip** ) )  AND  TITLE-ABS-KEY ( *rate** ) )

| # 3 | **53** | #2 AND #1  *Indexes=SCI-EXPANDED, SSCI, A&HCI, ESCI Timespan=All years* |
| --- | --- | --- |
| # 2 | **9,621,900** | **TOPIC:** (high*) *OR* **TOPIC:** (increas*) *AND* **TOPIC:** (respons*) *OR* **TOPIC:**   (particip*) *AND* **TOPIC:** (rate*)  *Indexes=SCI-EXPANDED, SSCI, A&HCI, ESCI Timespan=All years* |
| # 1 | **93** | **TOPIC:** ("health examination survey?")  *Indexes=SCI-EXPANDED, SSCI, A&HCI, ESCI Timespan=All years* |

**Database: PUBMED**

**Date:** 26.06.2017

**Hits:** 39

| #28, (#17 AND #27) Filters: Humans | 39 |
| --- | --- |
| #27,"High response rates"" Filters: Humans | 978 |
| #17, (#15 OR #16) | 459221 |
| #16, health examination survey[Title/Abstract | 415 |
| #15,health surveys[MeSH Terms] Sort by: Title",458968 | 458868 |
