# Supplementary material for: Local problem solving in the Portuguese health examination survey: a mixed method study
Source: Arch Public Health. 2022 Aug 24;80:198. doi: 10.1186/s13690-022-00939-7 (PMC9400230; doi:10.1186/s13690-022-00939-7)
Supplement: Supplementary file 2 — Additional file 2: Questionnaire for local survey team members. [file 13690_2022_939_MOESM2_ESM.docx]

## Additional file 2: questionnaire for local survey team members

1. **What is your role regarding INSEF?**

| **Local coordinator high level** |
| --- |
| **Local coordinator low level** |
| **Laboratory** |

1. **Now that INSEF is over, are you happy that you participated?**

| **Very unhappy** |
| --- |
| **A little unhappy** |
| **Neither happy nor unhappy** |
| **A little happy** |
| **Very happy** |

1. **Why are you unhappy about your participation?**
2. **Did you personally learn anything by taking part in INSEF?**

| **I didn't learn anything** |
| --- |
| **I learned a little** |
| **I learned a lot** |

1. **Did your organisation learn anything by taking part in INSEF?**

| **We didn't learn anything** |
| --- |
| **We learned a little** |
| **We learned a lot** |

1. **Would you (personally) do another round of INSEF in a few years?**

| **Yes, absolutely** |
| --- |
| **Yes, I think so** |
| **Maybe** |
| **Probably not** |
| **Absolutely not** |

1. **Why not?**
2. **Was it easy or hard to get your organisation to cooperate regarding INSEF?**

| **Very hard** |
| --- |
| **Hard** |
| **Neither hard nor easy** |
| **Easy** |
| **Very easy** |

1. **What made it hard to get your organisation to cooperate?**
2. **Would it be useful to have another INSEF in a few years?**

| **Yes, very useful** |
| --- |
| **Yes, somewhat useful** |
| **No, not useful** |

1. **At this INSEF, what were the most difficult things? (choose up to three)**

| **Getting local permission** |
| --- |
| **Getting local funding** |
| **Getting time to do it** |
| **Cooperating with health centres** |
| **Cooperating with INSA** |
| **Training personnel** |
| **Getting personnel** |
| **Practical problems regarding examination** |
| **Cooperating with labs** |
| **Other** |

1. **What can INSA do to facilitate another INSEF?**
2. **What can your organisation do to facilitate another INSEF?**
3. **Please describe a practical problem you faced and how it was solved**
4. **Please describe an organisational problem you faced and how it was solved**
5. **Please write here anything else you think we should know**
