# Supplementary material for: Local problem solving in the Portuguese health examination survey: a mixed method study
Source: Arch Public Health. 2022 Aug 24;80:198. doi: 10.1186/s13690-022-00939-7 (PMC9400230; doi:10.1186/s13690-022-00939-7)
Supplement: Supplementary file 3 — Additional file 3: Informal interview guide. [file 13690_2022_939_MOESM3_ESM.docx]

## Additional file 3: informal interview guide

What was your role in INSEF?

What were your tasks?

Who did you have to collaborate with to do your tasks?

Tell me more about this collaboration

Was it easy or hard to get the job done?

What made it easy/hard?

Was the manual sufficient to get the job done?

When it wasn’t – what did you do?

What happened?
