# Supplementary material for: Local problem solving in the Portuguese health examination survey: a mixed method study
Source: Arch Public Health. 2022 Aug 24;80:198. doi: 10.1186/s13690-022-00939-7 (PMC9400230; doi:10.1186/s13690-022-00939-7)
Supplement: Supplementary file 4 — Additional file 4: Supplementary materials. [file 13690_2022_939_MOESM4_ESM.docx]

## Additional file 4: supplementary materials

The following materials were also consulted, but as they did not directly represent the local health survey teams and their opinions, they were not coded.

- Copies of the INSEF newsletter7s (N1 –N6)
- The confidential site visit report from November 2015 by Hanna Tolonen and Päivikki Koponen from the Finnish Institute for Health and Welfare (THL)
- Assorted documentation from meetings between INSA and NIPH
- Fieldwork barometer (weekly newsletter sent to all the local teams) from weeks 16 and 32 (April and October, 2015)
- The article Fieldwork Monitoring Strategies in a Health Examination Survey [2] (corroborating some of the findings from the other materials, particularly regarding the way the local teams expanded opening hours to allow more invitees to participate in the health survey, and regarding the support the central team gave the local teams)
- Regional training reports
