# Supplementary material for: Local problem solving in the Portuguese health examination survey: a mixed method study
Source: Arch Public Health. 2022 Aug 24;80:198. doi: 10.1186/s13690-022-00939-7 (PMC9400230; doi:10.1186/s13690-022-00939-7)
Supplement: Supplementary file 5 — Additional file 5: Code list used in Thematic Analysis. [file 13690_2022_939_MOESM5_ESM.docx]

## Additional file 5: code list used in Thematic Analysis

1. Recruitment of professionals
2. Issues with leaders (and their (missing) involvement)
3. Financial issues
4. Blood transport
5. Internal organisational issues (bureaucracy)
6. Geographic accessibility
7. Dissemination (in media, to health centres etc.) before survey
8. Contact worksheet
9. Recruiting participants
10. Reserve working time (for professionals)
11. Problems with physical facilities
12. Transportation issues (for personnel and patients)
13. Longer survey period
14. Computer problems
15. Database not updated
16. Positive communication with INSA team
17. Negative internal collaboration within team
18. Positive internal collaboration within team
19. Working overtime
20. Informal strategies to solve problems
21. Laboratory problems re availability
22. Use of survey (show local authorities that a survey is useful)
23. Issues with health centres
24. Training
